# Subspecies Identification and Characterization of Drug Resistance and Virulence Factors in Clinical Strains of *Mycobacterium abscessus* Complex Isolated from South India

**DOI:** 10.3390/idr18040073

**Published:** 2026-07-13

**Authors:** Kumaran Oudhaya, Ellappan Kalaiarasan, Anoop Alex, Kooleri Padinjare Veetil Hyma, Harishni Padmanaban, Sangitha Jayagandan, Noyal Mariya Joseph

**Affiliations:** 1 Department of Microbiology, Jawaharlal Institute of Postgraduate Medical Education and Research, Puducherry 605 006, India; jr8072@jipmer.ac.in (K.O.); drkalaiarasan.e@gmail.com (E.K.); jr7317@jipmer.ac.in (K.P.V.H.); amul4hari@gmail.com (H.P.); sangitha.j.2018@gmail.com (S.J.); 2 CIIMAR/CIMAR, Interdisciplinary Centre of Marine and Environmental Research, Terminal de Cruzeiros de Leixões, Av. General Norton de Matos s/n, 4450-208 Matosinhos, Portugal; anoopthycaud@gmail.com; 3 Tezzonix Research, Bioinformatics & Computational Biology Division, Tezzonmaart Technology Ventures Pvt. Ltd., Kochi 686 691, India

**Keywords:** *Mycobacterium abscessus*, colony morphology, antimicrobial resistance, biofilms, whole-genome sequencing, South India

## Abstract

Background: *Mycobacterium abscessus* complex (MABC), comprising *Mycobacterium abscessus* subsp. *abscessus* (MABa), *Mycobacterium abscessus* subsp. *bolletii* (MABb), and *Mycobacterium abscessus* subsp. *massiliense* (MABm), is an emerging group of non-tuberculous mycobacteria with clinically significant infections and challenging treatment outcomes due to extensive antimicrobial resistance. Accurate subspecies identification and characterization of resistance- and virulence-associated determinants are essential for effective disease management. This study aimed to determine the prevalence and subspecies distribution of MABC and to characterize resistance-associated mutations and virulence factors, including biofilm formation. Methods: A total of 1110 NTM-suspected clinical samples were screened during the study period, between January 2024 and October 2025. Samples negative by GeneXpert MTB/RIF were subjected to Mycobacteria Growth Indicator Tube (MGIT) culture, followed by Ziehl–Neelsen staining and MPT64 antigen testing. Acid-fast bacilli-positive, MPT64-negative isolates were identified as NTM and analyzed using GenoType CM and NTM-DR line probe assays (LPA) for species identification and detection of resistance-associated mutations. A polymerase chain reaction (PCR) assay was optimized to differentiate MABa and MABm. All MABC clinical strains were further characterized for colony morphology (smooth and rough) and biofilm formation. Three biofilm-producing MABa strains (2 rough and 1 smooth) that were detected as macrolide-resistant by NTM-DR were subjected to whole-genome sequencing (WGS). Results: Among 1110 clinical samples, MABC was identified in 2.25% (*n* = 25) of cases, while other NTM species accounted for 4.41% (*n* = 49). Among 25 MABC clinical strains, 14 (56%) were MABm, and 11 (44%) were MABa, as confirmed by both LPA and PCR. LPA-NTM DR detected *erm(41)* T28 sequevar (*n* = 9) and C28 mutation (*n* = 2) among MABa strains, with one strain exhibiting aminoglycoside resistance-associated *rrs* mutation. Nineteen isolates displayed a smooth morphotype (MABa = 8 and MABm = 11), and six were rough (MABa = 3 and MABm = 3). Biofilm formation was observed in both smooth (*n* = 5) and rough (*n* = 4) morphotypes. WGS analysis confirmed *erm(41)* T28 sequevar, identified a missense mutation (A238G), and revealed genes associated with glycopeptidolipid biosynthesis. Conclusions: Our findings provide important insights into subspecies identification and genetic determinants associated with drug resistance and virulence in MABC. The biofilm-forming ability observed in both smooth and rough morphotypes emphasizes its potential role in persistence and treatment challenges, emphasizing the need for comprehensive diagnostic strategies.

## 1. Introduction

*Mycobacterium abscessus* complex (MABC) is one of the most clinically relevant and drug-resistant (DR) non-tuberculous mycobacteria (NTM) that cause severe nosocomial infections in patients with cystic fibrosis or immunocompromising conditions [1]. The *Mycobacterium abscessus* (MAB) genome (CIP 104536T) consists of a 5,067,172 bp circular chromosome with 4920 predicted coding sequences and a 23,319 bp plasmid [2]. Additionally, the MAB genome includes an 81-kilobase full-length prophage and five insertion sequences (IS) elements [2]. The MABC, which are responsible for 2.6–13.0% of all NTM pulmonary infections [1], have been classified into three subspecies, *Mycobacterium abscessus* subsp. *abscessus* (MABa), *Mycobacterium abscessus* subsp. *bolletii* (MABb), and *Mycobacterium abscessus* subsp. *massiliense* (MABm). Thangavelu et al. 2021, from South India, reported that the prevalence of NTM among pulmonary and extrapulmonary mycobacterial isolates was 10.5% and 8.4%, respectively. Furthermore, MABC accounted for 17.7% of NTM isolates recovered from pulmonary specimens [3]. Treating infections caused by MABC remains challenging due to intrinsic drug resistance to many antibiotics and potential adaptive and acquired resistance. MABC subspecies vary in macrolide susceptibility depending on the presence and functionality of the erythromycin ribosomal methylation gene 41 (*erm41*). MABa strains that carry a fully functional *erm(41)* gene result in inducible macrolide resistance, specifically clarithromycin resistance due to upregulation of *erm(41)*, which modifies the ribosomal RNA. The T/C polymorphism at position 28 of the *erm(41)* gene in MABa plays a vital role in macrolide resistance [4]. MABa strains with the T28 genotype are inducible resistant, while those with the C28 genotype are susceptible. MABm is mostly susceptible to macrolides, specifically clarithromycin, due to a non-functional *erm(41)* gene with a 271 bp deletion [5]. As extended incubation periods are needed to identify inducible macrolide susceptibility profiles phenotypically, molecular methods targeting the *erm(41)* gene can be used as a more reliable and alternative tool [4]. MABC can develop acquired drug resistance to aminoglycosides and macrolides through mutations in the *rrs* (16S ribosomal ribonucleic acid (rRNA)) and *rrl* (23S rRNA) genes, respectively [6]. Since MABC infections are increasing globally and drug susceptibility varies among their subspecies, clinicians should prioritize accurate identification of MABC in clinical settings to guide the appropriate therapy.

MABC exhibits two distinct colony morphotype variants on solid agar media, termed smooth (S) and rough (R). The S colonies are shiny and soft, and R colonies are dry, irregular/wrinkled, and non-shiny; smooth colonies are shiny and mucoid/waxy. The S morphotype expresses high levels of glycopeptidolipids (GPL) in its cell wall; in contrast, the R morphotype exhibits reduced GPL [7,8]. The transition from S to R morphotype in MABC is mostly associated with the genetic mutations in GPL biosynthesis or transport genes, which are involved in GPL synthesis and secretion [7,8]. MABC yields inimitable polar GPLs that are diglycosylated on alaninol and di-O-acetylated on 6-deoxytalose [9]. GPLs act as potential virulence factors by covering the bioactive cell wall lipids of MABC and delaying the activation of the host’s immune system during the early stages of colonization. This delay allows the MABC bacilli to establish themselves in the host before the complete activation of the immune response, which leads to successful infection [9,10].

Biofilm formation is one of the important aspects of MABCs’ pathogenicity. Bacteria in biofilms are enveloped in an extracellular matrix that is composed of lipids, proteins, extracellular DNA, and polysaccharides [11]. MABC can form biofilms in municipal water supplies and on hospital equipment, acting as a potential source of infection [12]. Both S and R morphotypes form biofilms in the lungs, which can provide antimicrobial resistance and may help the bacteria evade host immune responses [13]. It has been demonstrated that changes in colony morphotypes correlated with differences in the formation of biofilms and the mechanical properties of biofilms [14]. The *S variants* colonize abnormal lung airways in a biofilm, and a spontaneous reduction in GPL changes the colony morphology to an R type, causing inflammation and invasive disease [15,16]. The R colony type is known to form pellicular biofilms and is characterized by a higher degree of mechanical resistance, despite lacking GPL. Although studies have revealed that S and R morphotypes influence biofilm production in MABC infections, it is important to assess the other factors that enhance biofilm formation among such infections. In this context, based on the background knowledge, we aimed (i) to identify and profile the MABC subspecies using molecular techniques and (ii) to characterize antimicrobial resistance and virulence factors, including colony morphotypes and biofilm formation in MABC clinical strains isolated from a South Indian tertiary care hospital.

## 2. Methodology

### 2.1. Study Design and Ethical Considerations

This cross-sectional analytical study was conducted in the Department of Microbiology, Jawaharlal Institute of Postgraduate Medical Education and Research (JIPMER), Puducherry, India. This study was approved by the Institute Ethics Committee (Approval No. JIP/IEC-OS/265/2023—dated 3 October 2023), JIPMER, Puducherry, India.

### 2.2. Sample Collection and Bacterial Culture

A total of 1110 NTM-suspected clinical samples, comprising both pulmonary and extrapulmonary specimens, were collected during the study period (January 2024 and October 2025) and processed in accordance with the American Thoracic Society (ATS) guidelines for NTM disease [17]. A consecutive sampling strategy was adopted. NTM strains isolated from clinical specimens of patients suspected of NTM infection and subjected to routine diagnostic evaluation in the Department of Microbiology were included. Repeated clinical strains from the same patient were excluded from this study. Among these, MABC strains were selected for further analysis. All samples were initially screened using GeneXpert MTB/RIF to detect *Mycobacterium tuberculosis* complex (MTBC) [18]. Samples negative for MTBC were subjected to Mycobacteria Growth Indicator Tube (MGIT) culture, followed by Ziehl–Neelsen staining for detection of acid-fast bacilli [17,18,19]. AFB-positive cultures that tested negative for MPT64 antigen were classified as NTM. All consecutive MABC clinical strains recovered between January 2024 and October 2025 were included and identified using the GenoType Common Mycobacteria (CM) assay.

### 2.3. Line Probe Assay Genotype Mycobacterium CM (LPA-CM)

A total of 74 NTM MGIT cultures isolated from clinical samples were subjected to LPA-CM [19]. Briefly, MGIT cultures (1.5 ml) were centrifuged (10,000 rpm) for 10 min, the supernatant was discarded, and DNA was extracted from the pellet as per the manufacturer’s instructions (GenoLyse kit, Hain Lifescience GmbH, Nehren, Germany). The DNA was subjected to Polymerase Chain Reaction (PCR) using the Mastercycler (Eppendorf, Hamburg, Germany). The PCR-amplified products were subjected to reverse hybridization on nitrocellulose membrane strips to identify the mycobacterium species subsequently. Reverse hybridization was performed using TwinCubator (Hain Lifescience).

### 2.4. The GenoType NTM-DR Line Probe Assay (NTM-DR)

All 25 MABC cultures were subjected to NTM-DR as per the manufacturer’s instructions (Hain Lifescience) [20]. NTM-DR was used to identify subspecies, together with the detection of aminoglycosides (*rrs* gene) and macrolides [*erm(41)* and *rrl* genes] resistance genotypes. MGIT cultures were subjected to DNA extraction as per the manufacturer’s instructions (GenoLyse kit, Hain Lifescience). The extracted DNA was used for PCR amplification, followed by reverse hybridization on nitrocellulose membrane strips, and then developed and interpreted (Hain Lifescience).

### 2.5. DNA Extraction and PCR

A total of 25 MABC strains were subjected to DNA extraction using the Cetyltrimethylammonium-bromide (CTAB) method [21]. The extracted DNA was subjected to PCR assays targeting Cytochrome c oxidase subunit III (*CytIII*) and PadR family transcriptional regulator (*PadR*) to identify Mab subsp. *abscessus* (MABa) and Mab subsp. *massiliense* (MABm), respectively [22]. During the PCR assay, the 25 μL of total reaction mixture contained 12.5 μL of Master Mix (Promega, Madison, WI, USA), primers (1.5 μL, forward and 1.5 μL, reverse), 5 μL of DNA, and nuclease-free water (4.5 μL). The thermal conditions that are used to detect both the *CytIII* and *PadR* genes are as follows: 1 cycle of initial denaturation at 95 °C (3 min); 30 cycles of denaturation, 95 °C (30 s), annealing, 53 °C (30 s), and extension, 72 °C (30 s); and a final extension for 5 min at 72 °C (1 cycle). The amplified products were determined using 1.8% agarose gel electrophoresis. The primers used in this study are listed in Table 1. A MABa isolate positive for the *CytIII* target, and a MABm isolate positive for the *PadR* target were confirmed by sequencing, followed by sequence analysis using the Basic Local Alignment Search Tool (BLAST), National Center for Biotechnology Information (NCBI), Bethesda, MD, USA (http://www.ncbi.nlm.nih.gov/BLAST/) (accessed on 5 June 2025). The confirmed strains were used as positive controls (PCs) for subsequent PCR experiments. A negative control (NC) was run concurrently for each experiment.

### 2.6. Colony Morphology

The 25 MABC-positive MGIT cultures were inoculated on blood agar to identify the colony morphotypes. The MABC strains inoculated into blood agar were incubated for 5 days at 37 °C. The rough (R) strains exhibited a colony texture that was rough and dry, and the smooth (S) strains were smooth, shiny, and waxy [6].

### 2.7. Quantitative Biofilm Assays

All 25 MABC clinical strains were tested for biofilm formation using a 96-well microtiter plate (MTP) screening assay. A biofilm-producing MABa strain was used as a positive control. The three-day-old MGIT cultures of MABC were adjusted to 0.5 McFarland, diluted to achieve 5 × 10^6^ CFU/mL and incubated for 14 days in 7H9 broth supplemented with OADC supplement. After 14 days of incubation, the MTP was washed with distilled water rapidly and air-dried. The plate was then stained with crystal violet (0.2%) and kept at room temperature for 10 min, followed by washing with distilled water and air-drying [23]. Then, 95% of ethanol was added to the wells and kept at room temperature for 15 min. The absorbance was measured at 570 nm using a plate reader (BMG LABTECH, Ortenberg, Germany). The biofilm experiments were carried out in duplicates, and the mean optical density (OD) values were calculated.

### 2.8. Whole-Genome Sequencing (WGS)

#### 2.8.1. DNA Extraction and Quality Check

In this study, three biofilm-producing macrolide-resistant MABa strains (MAB1, MAB4, and MAB6) (detected by LPA-NTM DR) harboring the T28 sequevar of *erm(41)* were subjected to whole-genome sequencing (WGS). DNA extraction from the three MABa strains was performed using the CTAB method [21]. The concentration of the extracted DNA was measured using a Qubit Fluorometer (Thermo Fisher Scientific, Waltham, MA, USA) with the Qubit dsDNA Quantification Assay Kit (Cat. No. Q32854; Thermo Fisher Scientific, Waltham, MA, USA), and DNA integrity was determined by 0.8% agarose gel electrophoresis.

#### 2.8.2. DNA Library Preparation Protocol

The Twist EF Library PrepKit for Illumina (Cat. No. 100572; Twist Bioscience, South San Francisco, CA, USA) was used to prepare whole-genome libraries by performing fragmentation, end-repair, and monoadenylation in a single enzymatic reaction, followed by adapter ligation. Unique primers with barcodes were then used for PCR-based indexing to enable multiplexing. Fragment distribution was analyzed using a 5300 Fragment analyzer (Agilent Technologies, Santa Clara, CA, USA).

#### 2.8.3. Sequencing

The Qubit HS assay (Cat. No. Q32854; Thermo Fisher Scientific, Waltham, MA, USA) was used to quantify the prepared libraries. After quantification, the libraries were pooled and diluted to the optimal concentration, and sequenced at 2 × 150 bp reads (paired-end) on Illumina Nova Seq XPlus.

#### 2.8.4. Bioinformatics

The FastQ files generated were initially checked for read quality. The FastQ files were subjected to adaptor trimming using FastQ-MCF (v-1.04.803, Expression Analysis Inc., Durham, NC, USA) to retain high-quality sequences for further analysis. The trimmed reads were then mapped to *M. abscessus* ATCC 19977 for reference-guided assembly. The aligned reads were used to generate consensus FASTA files using SAMtools (version 1.2, Wellcome Sanger Institute, Hinxton, Cambridgeshire, UK) [24]. The bedtools (version 2.0, Quinlan Laboratory, University of Utah, Salt Lake City, UT, USA) and an in-house Perl script were used to calculate coverage and depth statistics from the aligned reads [24]. GATK HaplotypeCaller (version gatk-4.1.0.0, Broad Institute, Cambridge, MA, USA) was used for variant calling, and the variants were further annotated using SnpEff (version 5.2c, Pablo Cingolani, Detroit, Michigan, USA). Finally, the genome was annotated using Prokka (version 1.14.6, The University of Melbourne, Melbourne, Victoria, Australia), and functional annotation and COG assignments were performed using eggNOG mapper (emapper, version2.0.1, EMBL, Heidelberg, Germany), respectively [24,25].

### 2.9. Statistical Analysis

The overall distributions of subspecies, gene positivity, colony morphology, and biofilm data were presented with frequencies and percentages. The agreement between LPA and PCR in detecting MABa and MABm was measured using the Kappa factor. Fisher’s exact test was performed to compare colony morphology between MABa and MABm strains. Additionally, Fisher’s exact test was applied to compare biofilm production between MABa and MABm, and between smooth and rough morphotypes. The statistical software, SPSS (Version 21; IBM, Armonk, NY, USA), was used for statistical analysis.

## 3. Results

### 3.1. Species Identification

**Figure 1:**
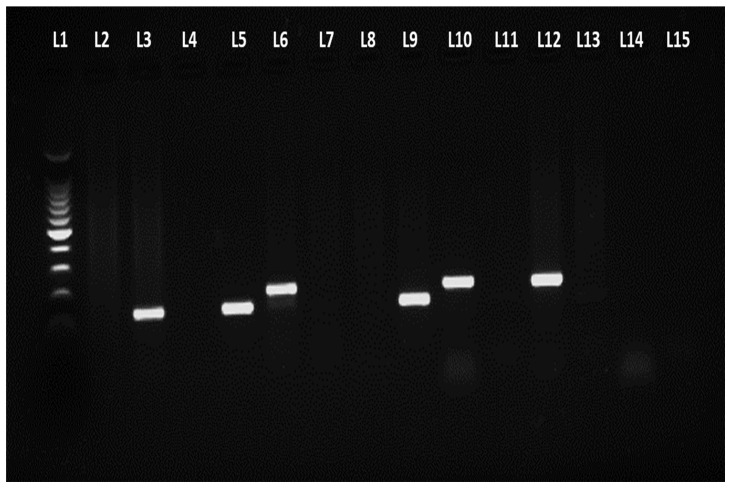
Identification of MABm and MABa strains using conventional PCR. PCR products were resolved on a 1.8% agarose gel. L1: 100 bp ladder. L3, L5, L9: amplification of *PadR* (142 bp) in MABm strains. L6, L10, L12: amplification of *CytIII* (190 bp) in MABa strains. L2, L4, L8: absence of *CytIII* genes in MABm. L7, L11, L13: absence of *PadR* genes in MABa strains. L14 and L15 are the negative controls for *CytIII* and *PadR*, respectively.

Of 1110 clinical samples, NTM strains were isolated from 74 (6.6%) samples by MGIT 960 culture. Among 74 NTMs, 25 clinical strains (33.7%) were identified as MABC, while the remaining 49 strains (66.2%) belonged to other NTM species. Among the 25 MABC clinical strains, the majority were obtained from pus samples (*n* = 13, 52%), followed by sputum (*n* = 4, 16%) and pleural fluid (*n* = 2, 8%). The remaining clinical strains were recovered individually from conjunctival swabs, drain fluid, cerebrospinal fluid, intraoperative specimens, soft palate tissue, and right percutaneous nephrostomy samples. MABC strains were predominantly isolated from male patients (*n* = 14, 56%), followed by female patients (*n* = 11, 44%). In this study, patients were categorized into six age groups (0–18, 19–30, 31–40, 41–50, 51–60, and >60 years). Among MABa clinical strains, the distribution across age groups was 0, 2, 1, 4, 2, and 2 clinical strains, respectively, whereas MABm strains were distributed as 1, 0, 2, 2, 5, and 4 strains, respectively. In addition, we also observed that MABm clinical strains were more frequently recovered from patients aged >50 years (64.3%, 9/14), whereas MABa strains were distributed across all age groups (Table 2). However, gender distribution did not differ significantly between subspecies (OR = 2.16, 95% CI: 0.430–10.845; *p* = 0.435) (Table 3a). Subspecies-level identification showed that both LPA (GenoType NTM-DR assay) and PCR detected 14 MABm clinical strains (56%) and 11 MABa (44%). The agreement between LPA and PCR was excellent, with a kappa value of 1.000. In PCR, the *PadR* target was detected in all MABm isolates, whereas the *CytIII* target was detected in all *MABa* isolates (Figure 1 and Table 2). In this current study, *Mycobacterium abscessus* subsp. *bolletii* (MABb) was not detected.

### 3.2. Detection of Antimicrobial Resistance in MABC Strains

Using the NTM-DR assay, antimicrobial resistance was assessed among the 25 MABC clinical strains. Among the 11 MABa isolates, nine (81.8%) were resistant to macrolides, while one isolate (9.1%) exhibited resistance to aminoglycosides. All nine macrolide-resistant MABa clinical strains harbored the T28 sequevar in the *erm(41)* gene, which is associated with inducible macrolide resistance. In addition, two MABa (18.2%) clinical strains were found to be carrying the C28 mutation in the *erm(41)* gene. No resistance to macrolides or aminoglycosides was detected among MABm isolates.

### 3.3. Colony Morphology and Biofilm Assays

**Figure 2:**
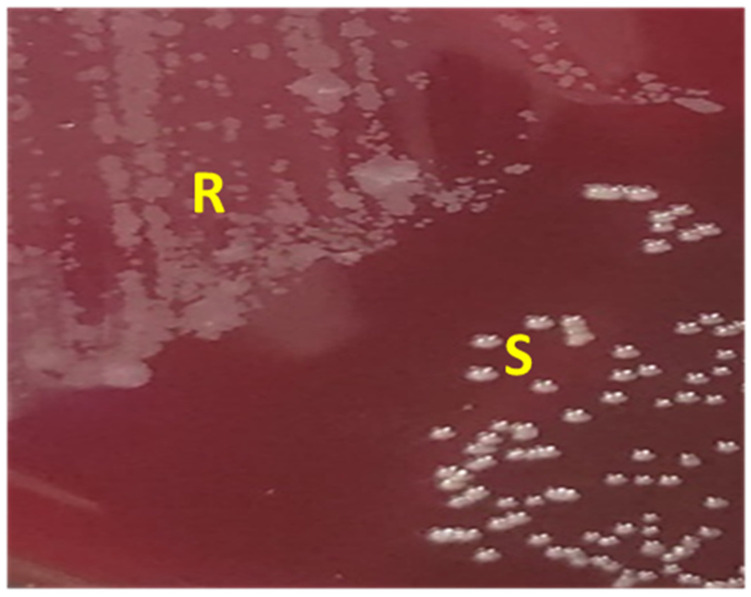
Representative colony morphologies; rough (R) and smooth (S). Rough colonies were dry and irregular; smooth colonies were shiny and waxy.

Among the 25 MABC clinical strains, 19 (76%) exhibited a smooth (S) colony morphology, while 6 (24%) showed a rough (R) morphotype (Figure 2). Among the smooth isolates, 11 (57.9%) were MABm, and 8 (42.1%) were MABa. Among the rough strains, 3 (50%) were MABm, and 3 (50%) were MABa. There was no statistically significant association between MABC subspecies and colony morphology (OR = 1.37, 95% CI: 0.218–8.669; *p* = 1.000) (Table 3b). Biofilm production among smooth morphotypes was observed in 5 of 19 clinical strains (26.3%), including 4 MABm and 1 MABa strains. In contrast, among rough morphotypes, biofilm production was detected in 4 of 6 clinical strains (66.7%), comprising 2 MABm and 2 MABa strains (Table 2). In addition, no statistically significant association was observed between biofilm production and MABC subspecies (OR = 2.00, 95% CI: 0.366–10.900; *p* = 0.677) (Table 3c). Although rough morphotypes showed a higher proportion of biofilm formation than smooth morphotypes, the association was not statistically significant (OR = 0.179, 95% CI: 0.025–1.294; *p* = 0.142) (Table 3d). 

### 3.4. Whole-Genome Sequencing

We sequenced the whole genomes of three biofilm-producing MABa strains (MAB1 (R), MAB4 (R), and MAB6 (S)) that were carrying the T28 sequevar of *erm(41)* (detected by LPA-NTM DR) on the Illumina NovaSeq XPlus platform. *Mycobacterium abscessus* ATCC 19977 was used as the reference genome for the data analysis. The alignment (%) and coverage (>=30X) of MAB1 with the reference genome were 95.38 and 91.16, respectively; MAB4 (95.86 and 92.22) and MAB6 (93.71 and 90.87). The average depth of MAB1, MAB4, and MAB6 was 88.29, 87.52, and 80.65, respectively. The information about total CDS, gene, rRNA, tRNA, and tmRNA for all three MABa strains was listed in the gene annotation table (Table S1, Supplementary Information). From WGS data, we observed that all three strains carried the T28 sequevar of *erm(41)* and a missense mutation in *erm(41)* (A238G). Both WGS and LPA-DR accurately identified the T28 sequence of *erm(41)*-associated inducible macrolide resistance. However, WGS that detected A238G missense mutations in *erm(41)* was not detected by LPA. Silent mutations, including T159C, G255A, and A330C, were also observed in all three strains. In addition, MAB4 and MAB6 strains were found to harbor G279T and T336C silent mutations in the *erm(41)* gene (Table 4). No mutations were observed in the *rrl* and *rrs* genes among the three MABa strains. In addition, all three strains harbored the *blaMAB* gene, which encodes a beta-lactamase responsible for hydrolyzing beta-lactams. In this study, WGS detected multiple point mutations in the genes associated with glycopeptidolipid (GPL) biosynthesis, including *Atf1*, *Atf2*, *mmpl4b*, *mmpl4a*, *gtf1*, *gtf2*, *gtf3*, *Sap*, *gap-like*, *rmt4*, and *FadE5* genes (Table 5). We detected mutations in both mmpl4a and mmpl4b genes of R strains, but not in S strains (Table 5).

The Clusters of Orthologous Groups (COG) functional analysis revealed that unknown functions were most prevalent across all three MABa strains. However, energy production and conversion, amino acid metabolism and transport, lipid metabolism and transport, cell wall/membrane/envelope biogenesis, inorganic ion transport and metabolism are the abundant functions in all three strains. But the gene counts in R strains (MAB1 and MAB4) were slightly higher compared to the S strain (MAB6) for lipid metabolism and transport, energy production and conversion, amino acid metabolism and transport, and secondary metabolites biosynthesis, transport, and catabolism (Figure S1a–c, Supplementary Information). Among 30 KEGG-enriched pathways, metabolic pathways, biosynthesis of secondary metabolites, and microbial metabolism in diverse environments were the top hits. The gene counts in metabolic pathways, biosynthesis of secondary metabolites, microbial metabolism in diverse environments, biosynthesis of antibiotics, and fatty acid degradation were slightly lower in the S strain as compared to the R strains (MAB1 and MAB4) (Figure S2a–c, Supplementary Information). The plots of the enriched Gene Ontology (GO) terms, including biological process, cellular component, and molecular function, were displayed in Figure S3a–c of the Supplementary Information.

## 4. Discussion

MABC is a rapidly growing and one of the most notorious causative agents of non-tuberculous mycobacterial (NTM) diseases and infections. It consists of three subspecies: MABa, MABb, and MABm [1,4]. Due to their different susceptibility patterns, these subspecies require rapid diagnosis and differentiation to select the appropriate therapy. Inducible resistance to macrolides is mostly observed in MABa and MABb strains mediated by *erm(41)*, while MABm does not possess inducible macrolide resistance due to a deletion in *erm(41)* [3,4,5]. The treatment of MABC infections remains challenging due to the availability of a limited number of antibiotics, extended treatment periods, and recurrent incidence of treatment-related toxicities. Hence, clinicians need to identify MABC subspecies together with drug resistance (DR). Line probe assays (LPAs), such as GenoType Mycobacterium CM (LPA-CM) and GenoType NTM-DR, are widely used for rapid identification of NTM species and detection of resistance to macrolides and aminoglycosides [19,20]. Several studies have demonstrated the utility of LPAs in routine diagnostics for NTM identification and DR profiling [26]. In the present study, 74 (6.6%) out of 1110 clinical samples were positive for NTM. Among these, 25 (33.7%) clinical strains were identified as MABC, while 49 (66.2%) belonged to other NTM species. Subspecies-level identification using the NTM-DR assay revealed 14 (56%) MABm and 11 (44%) MABa clinical strains. Analysis of the *erm(41)* gene revealed that nine MABa clinical strains harbored the T28 sequevar, while two isolates carried the C28 mutation. Notably, one MABa strain (MAB23) carrying the *erm(41)* C28 mutation, which is associated with macrolide susceptibility, exhibited aminoglycoside resistance. This finding is clinically important because aminoglycosides remain a cornerstone of *M. abscessus* treatment regimens, and resistance to this drug class may compromise therapeutic options despite preserved macrolide susceptibility. The *erm(41)* gene encodes a methyltransferase that modifies the bacterial ribosome (23S rRNA), thereby conferring resistance to macrolides [20,27]. The presence of the T28 sequevar results in a functional *erm(41)* protein, leading to inducible macrolide resistance. In contrast, the C28 mutation produces a non-functional protein, rendering the strain susceptible to macrolides [3,4]. In MABm, the *erm(41)* gene is typically truncated and non-functional, which explains its susceptibility to macrolides such as clarithromycin [5]. Although *erm(41)* plays a crucial role in resistance mechanisms, it is not subspecies-specific and therefore cannot be reliably used for subspecies classification [28]. To overcome this limitation, we employed PCR assays targeting the *PadR* and *CytIII* genomic targets to identify MABm and MABa, respectively, as described by Li et al. [22]. PCR results showed complete concordance with the LPA NTM-DR assay, identifying 14 (56%) MABm and 11 (44%) MABa clinical strains, with an excellent agreement (κ = 1.000). In our study setting in South India, MABm was more frequently isolated than MABa. This observation is consistent with reports from the UK, Europe, Australia, and the United States, where MABm has been reported to be highly prevalent, particularly among patients with cystic fibrosis [29,30]. In a multicenter study conducted across India (2021–2024), MABm accounted for 25 of 56 (44.6%) MABC isolates, while MABa accounted for 29 (51.8%) isolates, demonstrating the substantial contribution of MABm to MABC infections in India [31].

In addition to speciation and DR assays, we assessed colony morphology and biofilm production among 25 MABC strains. Colony morphology was evaluated on blood agar, and biofilm formation was measured using a 96-well microtiter plate assay, followed by crystal violet staining. On blood agar, 19 (76%) MABC strains exhibited smooth (S) morphology, whereas 6 (24%) displayed rough (R) morphology. Among the S morphotypes, 11 (57.9%) were MABm, and 8 (42.1%) were MABa. In contrast, among the R morphotypes, 3 (50%) were MABm and 3 (50%) were MABa. No statistically significant association was observed between MABC subspecies and colony morphology. The S and R morphotypes in MABC are distinguished by the presence or absence of glycopeptidolipids (GPL) on their cell surfaces [7,8,9]. The transition from S to R suggests that R strains may survive better in the host environment, increase resistance to phagocytosis, and be associated with more virulent infections [8]. Furthermore, biofilm formation by MABC has been linked to colony morphology, with R and S variants displaying different biofilm characteristics [14]. Such biofilm aggregates, often found in the lungs of patients with cystic fibrosis, show increased tolerance to various antibiotics. The formation of biofilms in clinical settings remains challenging, complicating treatment. Previous studies have reported that S morphotypes of MABC are typically associated with biofilm formation and reduced invasiveness, whereas R morphotypes are considered more invasive and less proficient in biofilm formation [32,33]. However, other studies have shown that R morphotypes can also form biofilms [34,35], consistent with our findings. Our study demonstrated biofilm formation in both S and R morphotypes. Among the 19 S clinical strains, 5 (26.3%) formed biofilms, including 4 MABm and 1 MABa. Notably, a higher proportion of R strains exhibited biofilm formation, with 4 out of 6 (66.7%) strains producing biofilms, comprising 2 MABm and 2 MABa (Table 2).

The three biofilm-producing MABa strains, identified as macrolide-resistant by LPA-NTM-DR, were subjected to whole-genome sequencing (WGS). Among the three MABa strains, two were R (MAB1 and MAB4), and one was S (MAB6). WGS results revealed that all three MABa strains carried the T28 sequevar of *erm(41)* and harbored a missense point mutation (A238G) and silent mutations (T159C, G255A, and A330C) in the *erm(41)* gene (Table 4). Although we identified an A238G missense mutation in *erm(41)* of MABa strains, further investigation is required to determine whether this single amino acid change affects the structure of the Erm protein. In addition, MAB4 and MAB6 strains were found to harbor G279T and T336C silent mutations in the *erm(41)* gene. Similarly, several studies have reported missense mutations (T28C, G76A, G158A, A238G, and C419T) and silent mutations (A120G, T159C, G168C, G255A, G279T, A330C, and T336C) in the *erm(41)* gene, as well as the A2059G mutation in the *rrl* gene, which are associated with macrolide resistance in the MABC [36,37]. The other mechanism of macrolide resistance, specifically clarithromycin resistance, is acquired through mutations in the 23S rRNA (*rrl*) gene at nucleotide positions 2058 and 2059, including A2058G/C and/or A2059G [38]. In WGS, we found that none of the three strains carried mutations in the *rrl* gene. In addition, we did not notice any mutations in the *rrs* gene, which is associated with aminoglycoside resistance. The GenoType NTM-DR assay results corroborated the WGS findings, as none of the three analyzed isolates (MAB1, MAB4, and MAB6) carried mutations in the rrl or rrs genes. We also observed that all three MABa strains harbored *blaMAB*. The *blaMAB* gene encodes β-lactamase enzyme in MABC, which can hydrolyze β-lactams and play a major role in the mycobacterial resistance to carbapenem antibiotics. Studies have identified that inhibiting *blaMAB* using β-lactamase inhibitors such as avibactam and vaborbactam can improve the effectiveness of β-lactam antibiotics against MABC [39,40]. Future studies on β-lactamase inhibitors may significantly enhance carbapenem activity against MABC infections. During WGS, we also found multiple point mutations in GPL biosynthesis-related genes (*Atf1*, *Atf2*, *mmpl4b*, *mmpl4a*, *gtf1*, *gtf2*, *gtf3*, *FadE5*, *Sap*, *gap*-like, and *rmt4*) [41,42] of S and R MABa strains (Table 5). In MABC strains, specifically MABm and MABa, the S and R morphotypes on solid agar are associated with the variations in glycopeptidolipid (GPL) production. The S morphotype produces high levels of GPLs, whereas the R morphotype has reduced GPL levels due to mutations in genes responsible for GPL synthesis. In the present study, we found that both of the R strains harbor mutations in mmpl4a and mmpl4 b genes, and not in the S strain. It has been reported that the mmpL4a-mmpL4b complex in MABC plays a crucial role in the biosynthesis and transport of GPLs [42]. These lipids are important for the bacteria’s surface properties, virulence, and morphology. The mmpL4a-mmpL4b proteins are membrane proteins encoded by genes that are organized in an operon with mmpS4 [43]. Disruption of these genes affects GPL production, leading to a change in colony morphology from smooth to rough. In our study, no mutations were observed in the mmps4 gene (MAB_4117c) in either the R or S strain. However, we detected multiple point mutations in the *Atf1* and *Atf2* genes of both S and R strains. *Atf1* and *Atf2* genes, which encode two putative O-acetyltransferases located within the GPL biosynthetic locus, transfer acetyl groups to 6-deoxy-α-L-talose [44]. In *Atf2*, the mutations C837T and T1904C were observed in all three strains, whereas C471A and C528G, and G334A and C969T were observed in MAB4 (R) and MAB6 (S), respectively. But studies also revealed that mutations or deletions in the *Atf1* and *Atf2* genes did not alter the GPL acetylation profile. Although *Atf1* and *Atf2* mutations do not influence colony morphology or GPL acetylation, they can affect the bacteria’s ability to survive within macrophages [44]. In MABC, the *gtf1*, *gtf2*, and *gtf3* genes play a crucial role in the synthesis of GPLs, which are complex glycolipids with varying structures. The *gtf1* and *gtf2* genes are involved in the early glycosylation steps of GPL biosynthesis, while *gtf3* adds a specific rhamnose residue at a later stage. These genes influence the composition and overall levels of GPLs, which in turn affect the bacterium’s colonial morphology and interactions with host cells. Studies have reported that mutations or deletions in the *gtf1*, *gtf2*, and *gtf3* genes may be associated with S-to-R conversion and virulence [45,46]. In our study, we noticed point mutations in *gtf1* of MAB1 (R) and MAB6 (S) and not in MAB4 (R), and *gtf2* of MAB4 (R) and MAB6 (S) and not in MAB1 (R). However, both R strains (MAB1 (R) and MAB4 (R)) harbor the same type of mutations, T1137C in *gtf3*. From these results, we observed that although mutations were identified in genes associated with GPL biosynthesis, other factors may also contribute to the observed morphological changes. In this study, WGS was performed on a small number of selected clinical strains, which may constrain the broader generalizability of the genomic findings.

## 5. Limitations

The present study has two major limitations. First, whole-genome sequencing was performed only on a limited number of isolates (biofilm-producing drug-resistant MABa). Second, although in-house phenotypic antimicrobial susceptibility testing for amikacin and clarithromycin was performed on selected MABC isolates, only validated genotypic resistance results generated using the GenoType NTM-DR assay were included due to methodological limitations associated with the in-house phenotypic assays.

## 6. Conclusions

The results of our study highlight the importance of the line probe assays (LPA) for the rapid and accurate identification of MABC subspecies and associated drug-resistance genotypes. A high proportion of MABa strains were found to be macrolide-resistant, predominantly harboring the T28 sequevar. Notably, two MABa clinical strains were macrolide-susceptible with the C28 mutation, of which one exhibited resistance to aminoglycosides. PCR assays targeting the *CytIII* and *PadR* genes could be a useful alternative for differentiating MABa and MABm in TB-endemic regions where LPA and whole-genome sequencing facilities are not readily available. Furthermore, both smooth and rough morphotypes exhibited the ability to form biofilms. Whole-genome sequencing analysis revealed genetic variations associated with antimicrobial resistance and virulence, especially in genes involved in glycopeptidolipid biosynthesis, which may contribute to the transition from smooth to rough morphotypes. This study underscores the value of subspecies-level identification and genomic analysis of MABC strains for understanding resistance mechanisms and informing future diagnostic and treatment strategies.

## Figures and Tables

**Table 1:** Primer used in the PCR assay for the detection of MABC subspecies.

Species	Gene	Direction	5′ to 3′	Amplicon Size (bp)	Reference
MABa	*CytIII*	Forward	CTTTGAATACGGTCGCCATCTGAC	190	[22]
		Reverse	GATACCTTCCAGTAGAGCTACGCC		
MABm	*PadR*	Forward	GAGAAGACACTGGCCCGATTCA	142	[22]
		Reverse	TGGTTCCTTCCTTACGGTCTTGAG		

**Table 2:** Identification of subspecies, drug resistance and virulence factors in MABC strains.

S. No	Lab No.	Sample	AGE	Gender	Species	Subspecies	PCR	(T28/C28)	(AMG) *	Morphology	Biofilm
1	MAB1	SPUTUM	48	F	*M. abscessus*	*abscessus*	*CytIII*	T28	Sensitive	Rough	Yes
2	MAB2	PUS	32	M	*M. abscessus*	*massiliense*	*PadR*	NA	Sensitive	Smooth	Yes
3	MAB3	PUS	64	M	*M. abscessus*	*massiliense*	*PadR*	NA	Sensitive	Smooth	No
4	MAB4	SPUTUM	64	M	*M. abscessus*	*abscessus*	*CytIII*	T28	Sensitive	Rough	Yes
5	MAB5	CONJUCTIVAL SWAB	61	M	*M. abscessus*	*massiliense*	*PadR*	NA	Sensitive	Smooth	Yes
6	MAB6	SPUTUM	54	F	*M. abscessus*	*abscessus*	*CytIII*	T28	Sensitive	Smooth	Yes
7	MAB7	PCN-Right	57	F	*M. abscessus*	*massiliense*	*PadR*	NA	Sensitive	Smooth	Yes
8	MAB8	PUS	7	M	*M. abscessus*	*massiliense*	*PadR*	NA	Sensitive	Rough	Yes
9	MAB9	PUS	52	F	*M. abscessus*	*massiliense*	*PadR*	NA	Sensitive	Smooth	No
10	MAB10	PLEURAL FLUID	45	M	*M. abscessus*	*massiliense*	*PadR*	NA	Sensitive	Smooth	Yes
11	MAB11	PUS	56	F	*M. abscessus*	*abscessus*	*CytIII*	T28	Sensitive	Rough	No
12	MAB12	PLEURAL FLUID	57	F	*M. abscessus*	*massiliense*	*PadR*	NA	Sensitive	Rough	Yes
13	MAB13	DRAIN FLUID	50	F	*M. abscessus*	*abscessus*	*CytIII*	T28	Sensitive	Smooth	No
14	MAB14	CSF	69	M	*M. abscessus*	*massiliense*	*PadR*	NA	Sensitive	Smooth	No
15	MAB15	INTRAOP	65	M	*M. abscessus*	*massiliense*	*PadR*	NA	Sensitive	Smooth	No
16	MAB16	PUS	37	M	*M. abscessus*	*abscessus*	*CytIII*	T28	Sensitive	Smooth	No
17	MAB17	PUS	55	M	*M. abscessus*	*massiliense*	*PadR*	NA	Sensitive	Smooth	No
18	MAB18	PUS	50	M	*M. abscessus*	*abscessus*	*CytIII*	T28	Sensitive	Smooth	No
19	MAB19	SPUTUM	56	F	*M. abscessus*	*massiliense*	*PadR*	NA	Sensitive	Smooth	No
20	MAB20	SOFT PALATE	24	M	*M. abscessus*	*abscessus*	*CytIII*	T28	Sensitive	Smooth	No
21	MAB21	PUS	40	M	*M. abscessus*	*massiliense*	*PadR*	NA	Sensitive	Smooth	No
22	MAB22	PUS	29	F	*M. abscessus*	*abscessus*	*CytIII*	C28	Sensitive	Smooth	No
23	MAB23	PUS	62	F	*M. abscessus*	*abscessus*	*CytIII*	C28	Resistant	Smooth	No
24	MAB24	PUS	42	M	*M. abscessus*	*abscessus*	*CytIII*	T28	Sensitive	Smooth	No
25	MAB25	PUS	45	F	*M. abscessus*	*massiliense*	*PadR*	NA	Sensitive	Rough	No

* AMG: Aminoglycoside resistance marker.

**Table 3:** (**a**): Fisher’s exact test to compare gender between MAB subspecies. (**b**): Fisher’s exact test to compare colony morphotypes between MABa and MABm. (**c**): Fisher’s exact test to compare biofilm production between MABa and MABm. (**d**): Fisher’s exact test to compare biofilm production between smooth and rough morphotypes.

(**a**)						
	MABm	MABa	*p* value	Odds ratio	95% Confidence interval	
					Lower	Upper
F	5	6	0.435	2.16	0.430	10.845
M	9	5				
(**b**)						
	MABm	MABa	*p* value	Odds ratio	95% Confidence interval	
					Lower	Upper
Smooth	11	8	1.000	1.37	0.218	8.669
Rough	3	3				
(**c**)						
	MABm	MABa	*p* value	Odds ratio	95% Confidence interval	
					Lower	Upper
Biofilm	6	3	0.677	2.0	0.366	10.9
Non-Biofilm	8	8				
(**d**)						
	Biofilm	Non-Biofilm	*p* value	Odds ratio	95% Confidence interval	
					Lower	Upper
Smooth	5	14	0.142	0.179	0.025	1.294
Rough	4	2				

**Table 4:** Detection of mutations in drug resistance-associated genes (macrolides and aminoglycosides) in S and R MABa—WGS.

S. No	Gene	Description	Mutations		
			* MAB1 (R)	* MAB4 (R)	* MAB6 (S)
1	*erm(41)*	methyltransferase	A238G T159C G255A A330C	A238G T159C G255A A330C G279T T336C	A238G T159C G255A A330C G279T T336C
2	*rrl*	23S ribosomal RNA	-	-	-
3	*rrs*	16S ribosomal RNA	-	-	-

* All the three strains (MAB1, MAB4 and MAB6) were found to be harbors of T28 sequevar.

**Table 5:** Detection of mutations in the GPL biosynthesis-related genes of R and S MABa-WGS.

S. No	Gene (Gene ID)	Description	Mutations		
			MAB1 (R)	MAB4 (R)	MAB6 (S)
1	*Atf1* (MAB_4106c)	Acetyltransferase	T288C	C135T C947G	A857C C947G
2	*Atf2* (MAB_4110c)	Acetyltransferase	C837T T1904C	C471A C528G C837T T1904C	G334A C837T C969T T1094C
3	*mmpl4b* (MAB_4115c)	RND family transporter	G1317A A1674G C2718G G2928C	C2250G	-
4	*mmpl4a* (MAB_4116c)	RND family transporter	G63C T378C G510A A2253G G2313A G2676A	C1767T	-
5	*gtf1* (MAB_4107c)	Glycosyltransferase GtfA	C247T C250T	-	G175C G808A
6	*gtf2* (MAB_4104)	Putative glycosyltransferase GtfB	-	A357G T939C A945G C1205T	C315T A357G T939C A945G C960T C1205T
7	*gtf3* (MAB_4112c)	Putative glycosyltransferase GtfA	G1014A T1137C	A348G T1137C	A348G
8	*FadE5* (MAB_4437)	Probable acyl-CoA dehydrogenase FadE	C534T G1589A C1734T	C534T G1589A	C534T G1589A
9	*mmpS4* (MAB_4117c)	MmpS family transport accessory protein	-	-	-
10	*Sap* (MAB_4454c)	YciI family protein	-	231G>A 225C>T 176A>G	231G>A 225C>T 176A>G
11	*gap*-like (MAB_4097c )	GAP family protein	-	T432C A468C	A468C
12	*gap*-like (MAB_0934)	GAP family protein	-	C150T C174T T225C C390T	C150T C174T T225C C390T
13	*rmt4* (MAB_4108c)	TylF/MycF family methyltransferase	-	A488G A450G	A488G A450G

## Data Availability

The original contributions presented in this study are included in the article and Supplementary Materials. Further inquiries can be directed to the corresponding author.

## Supplementary Materials

The following supporting information can be downloaded at: https://www.mdpi.com/article/10.3390/idr18040073/s1, Table S1: Genome Annotation Table; Figure S1: (a–c): COG functional classification of genes; Figure S2: (a–c): Top 30 KEGG enriched pathways; Figure S3: (a–c): Plot of the enriched GO terms.
