# Supplementary material for: Subspecies Identification and Characterization of Drug Resistance and Virulence Factors in Clinical Strains of Mycobacterium abscessus Complex Isolated from South India
Source: Infect Dis Rep. 2026 Jul 13;18(4):73. doi: 10.3390/idr18040073 (PMC13398286; doi:10.3390/idr18040073)
Supplement: Supplementary file 1 [file idr-18-00073-s001.zip › idr-4312526-supplementary.pdf]

Supplementary Information

Table S1: Genome Annotation Table

Strain no	Morphology	CDS	Gene	rRNA	tRNA	tmRNA
MAB1	Rough	4569	4620	3	47	1
MAB4	Rough	4600	4651	3	47	1
MAB6	Smooth	4558	4609	3	47	1

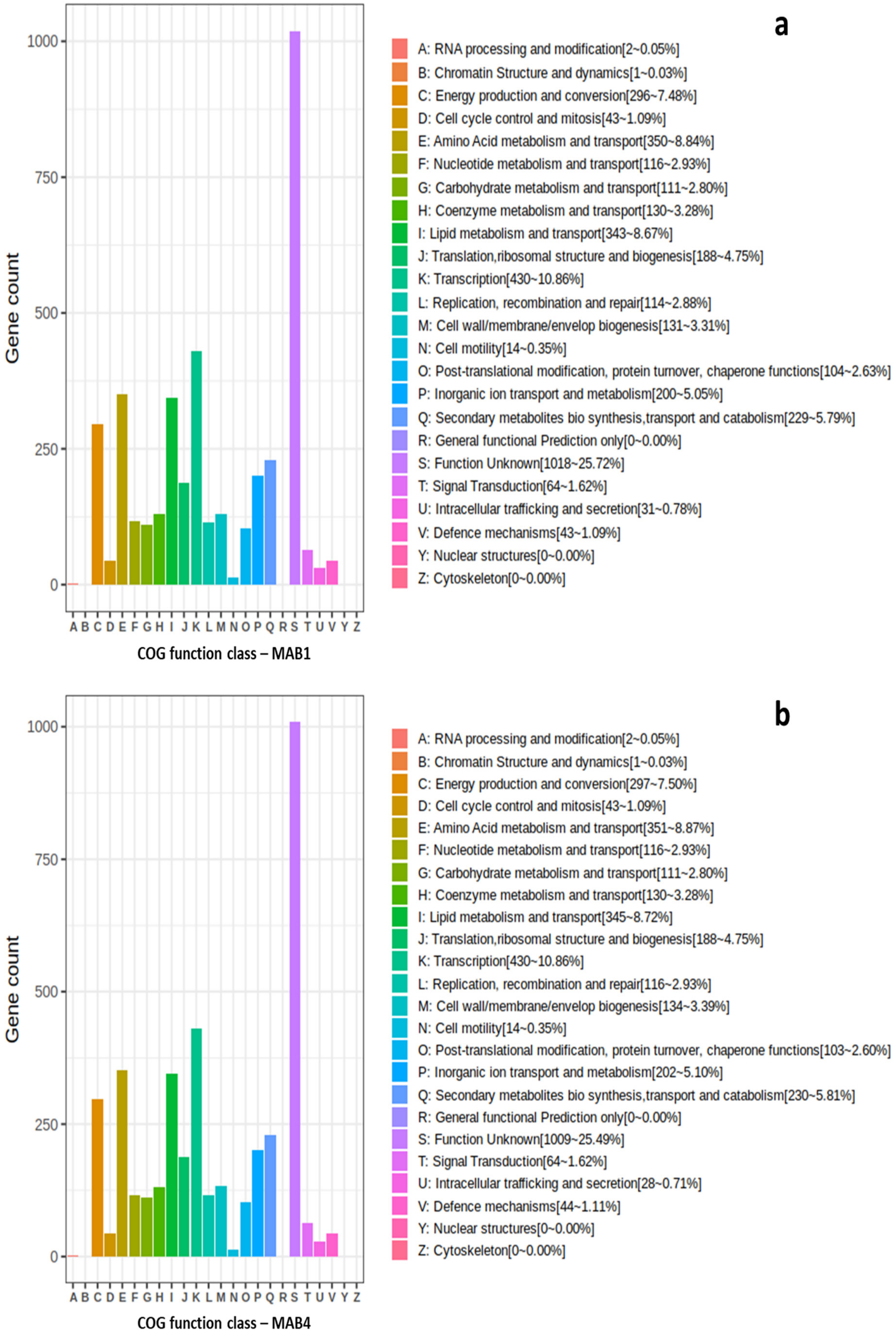
a
Gene count
COG function class – MAB1
A: RNA processing and modification[2~0.05%]
B: Chromatin Structure and dynamics[1~0.03%]
C: Energy production and conversion[296~7.48%]
D: Cell cycle control and mitosis[43~1.09%]
E: Amino Acid metabolism and transport[350~8.84%]
F: Nucleotide metabolism and transport[116~2.93%]
G: Carbohydrate metabolism and transport[111~2.80%]
H: Coenzyme metabolism and transport[130~3.28%]
I: Lipid metabolism and transport[343~8.67%]
J: Translation,ribosomal structure and biogenesis[188~4.75%]
K: Transcription[430~10.86%]
L: Replication, recombination and repair[114~2.88%]
M: Cell wall/membrane/envelop biogenesis[131~3.31%]
N: Cell motility[14~0.35%]
O: Post-translational modification, protein turnover, chaperone functions[104~2.63%]
P: Inorganic ion transport and metabolism[200~5.05%]
Q: Secondary metabolites bio synthesis,transport and catabolism[229~5.79%]
R: General functional Prediction only[0~0.00%]
S: Function Unknown[1018~25.72%]
T: Signal Transduction[64~1.62%]
U: Intracellular trafficking and secretion[31~0.78%]
V: Defence mechanisms[43~1.09%]
Y: Nuclear structures[0~0.00%]
Z: Cytoskeleton[0~0.00%]
b
Gene count
COG function class – MAB4
A: RNA processing and modification[2~0.05%]
B: Chromatin Structure and dynamics[1~0.03%]
C: Energy production and conversion[297~7.50%]
D: Cell cycle control and mitosis[43~1.09%]
E: Amino Acid metabolism and transport[351~8.87%]
F: Nucleotide metabolism and transport[116~2.93%]
G: Carbohydrate metabolism and transport[111~2.80%]
H: Coenzyme metabolism and transport[130~3.28%]
I: Lipid metabolism and transport[345~8.72%]
J: Translation,ribosomal structure and biogenesis[188~4.75%]
K: Transcription[430~10.86%]
L: Replication, recombination and repair[116~2.93%]
M: Cell wall/membrane/envelop biogenesis[134~3.39%]
N: Cell motility[14~0.35%]
O: Post-translational modification, protein turnover, chaperone functions[103~2.60%]
P: Inorganic ion transport and metabolism[202~5.10%]
Q: Secondary metabolites bio synthesis,transport and catabolism[230~5.81%]
R: General functional Prediction only[0~0.00%]
S: Function Unknown[1009~25.49%]
T: Signal Transduction[64~1.62%]
U: Intracellular trafficking and secretion[28~0.71%]
V: Defence mechanisms[44~1.11%]
Y: Nuclear structures[0~0.00%]
Z: Cytoskeleton[0~0.00%]

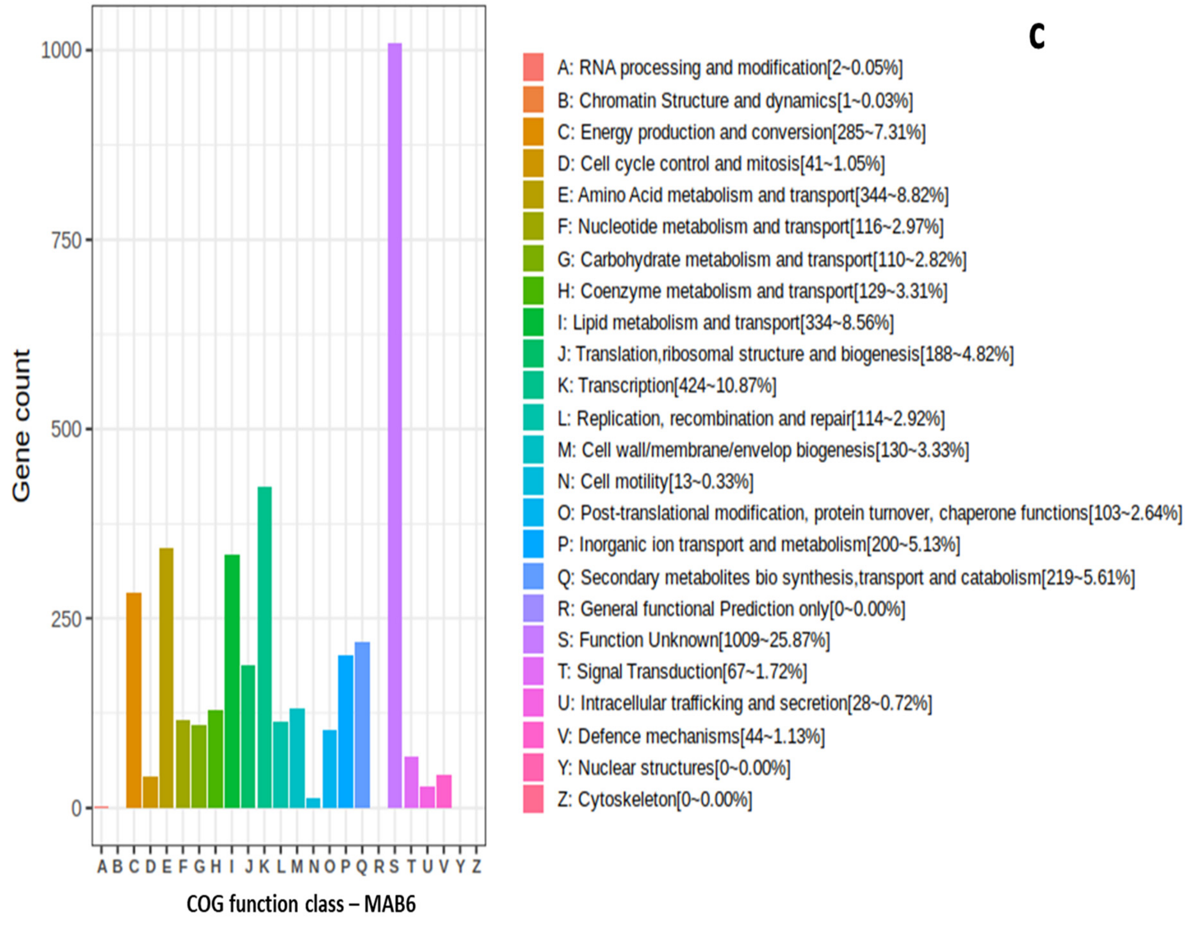

Figure S1 (a), (b) and (c): COG functional classification of genes. The function unknown category [S] had the highest number of genes. Other highly abundant COG functional categories included [C] Energy production and conversion, [E] Amino acid transport and metabolism, [I] Lipid transport and metabolism, [M] Cell wall/membrane/envelope biogenesis, and [P] Inorganic ion transport and metabolism.

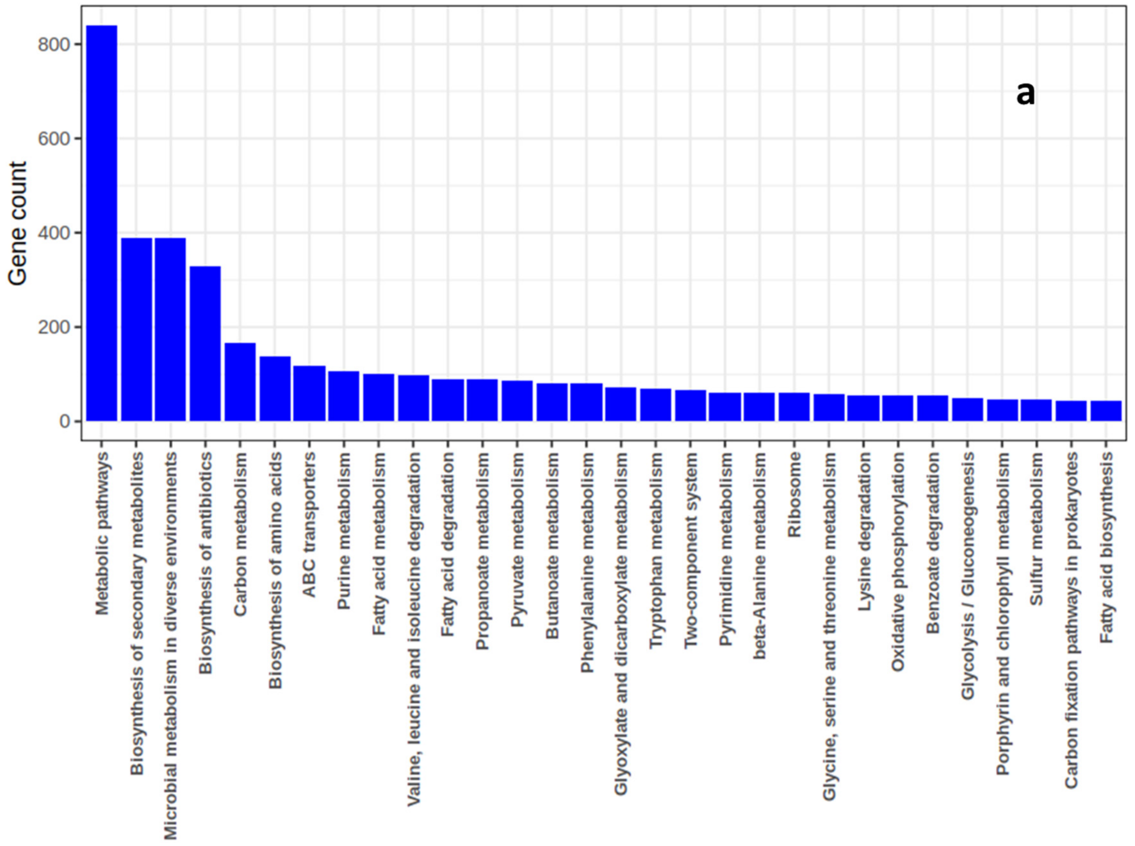

KEGG Pathways– MAB1

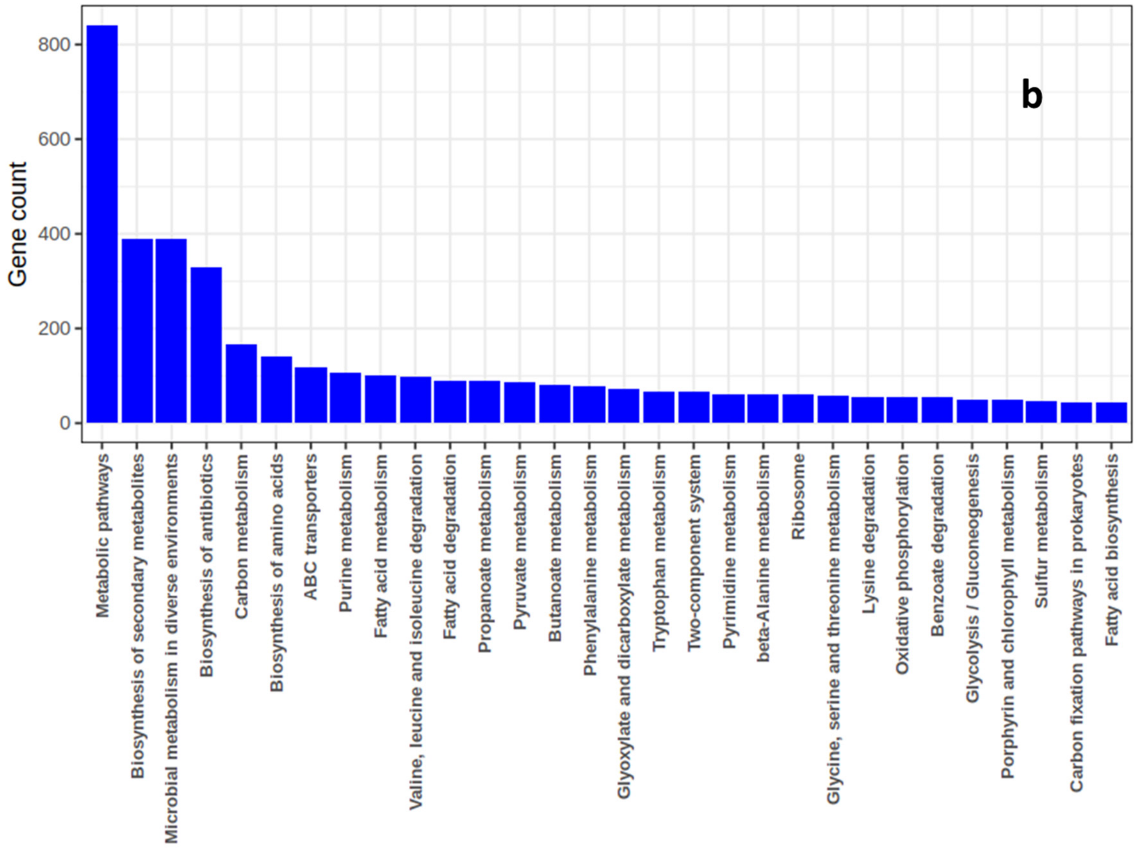

KEGG Pathways– MAB4

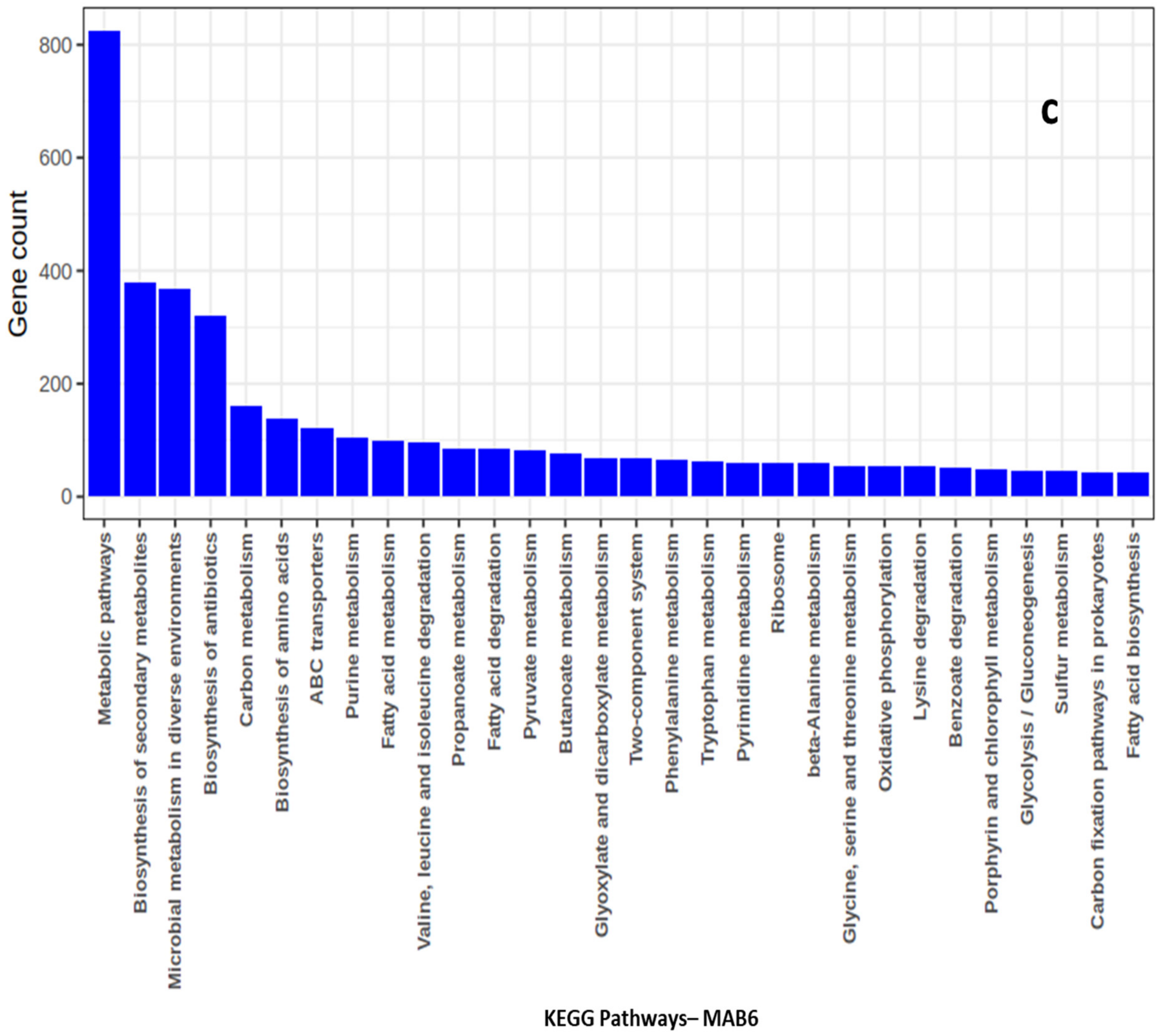

Figure S2 (a), (b) and (c): Top 30 KEGG enriched pathways. Metabolic pathways, Biosynthesis of secondary metabolites and Microbial metabolism in diverse environments were the top 3 hits.

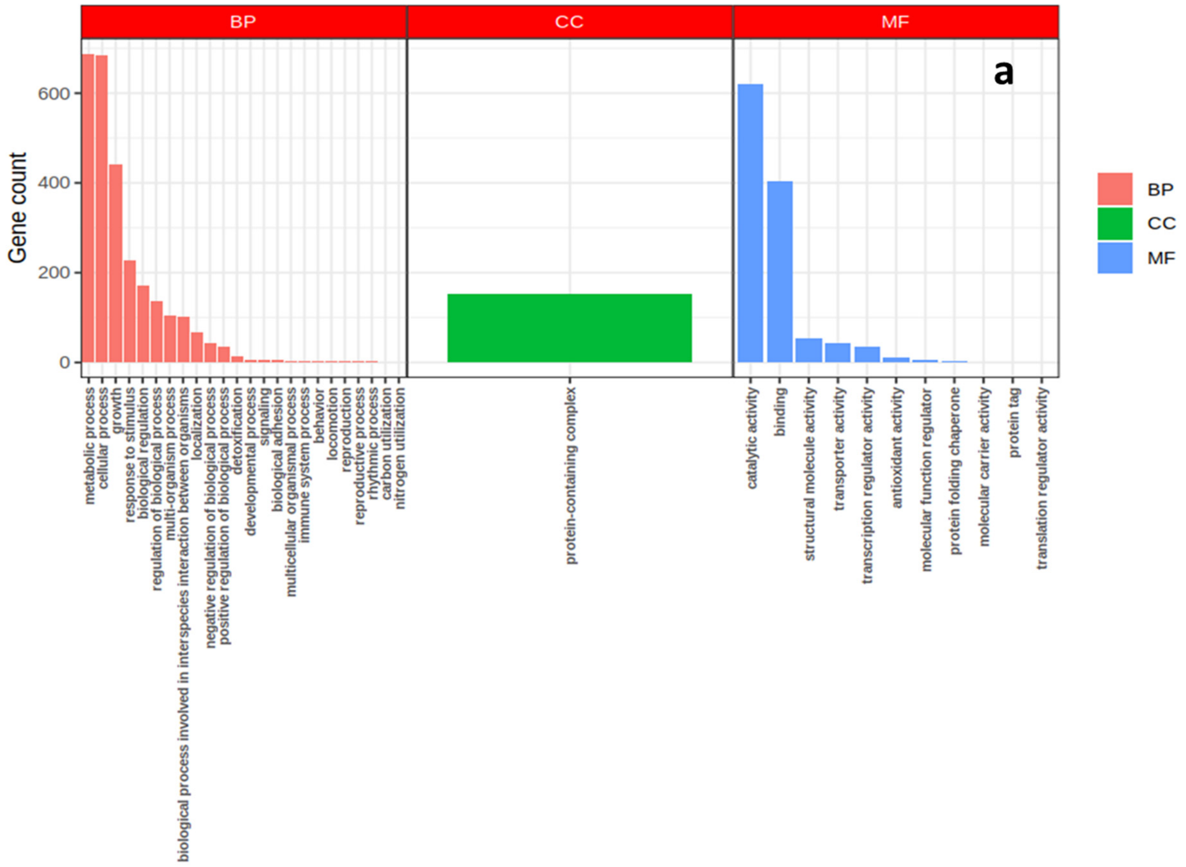

GO term values – MAB1

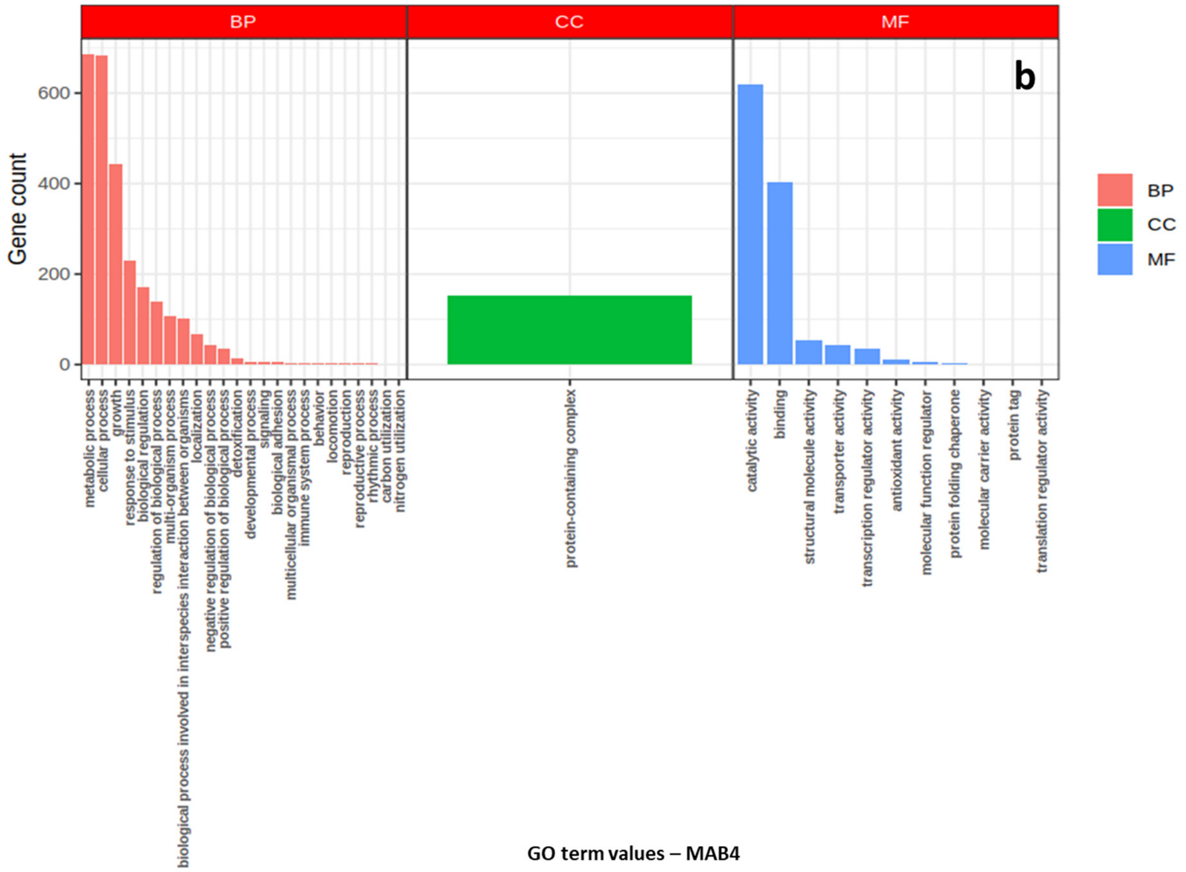

GO term values – MAB4

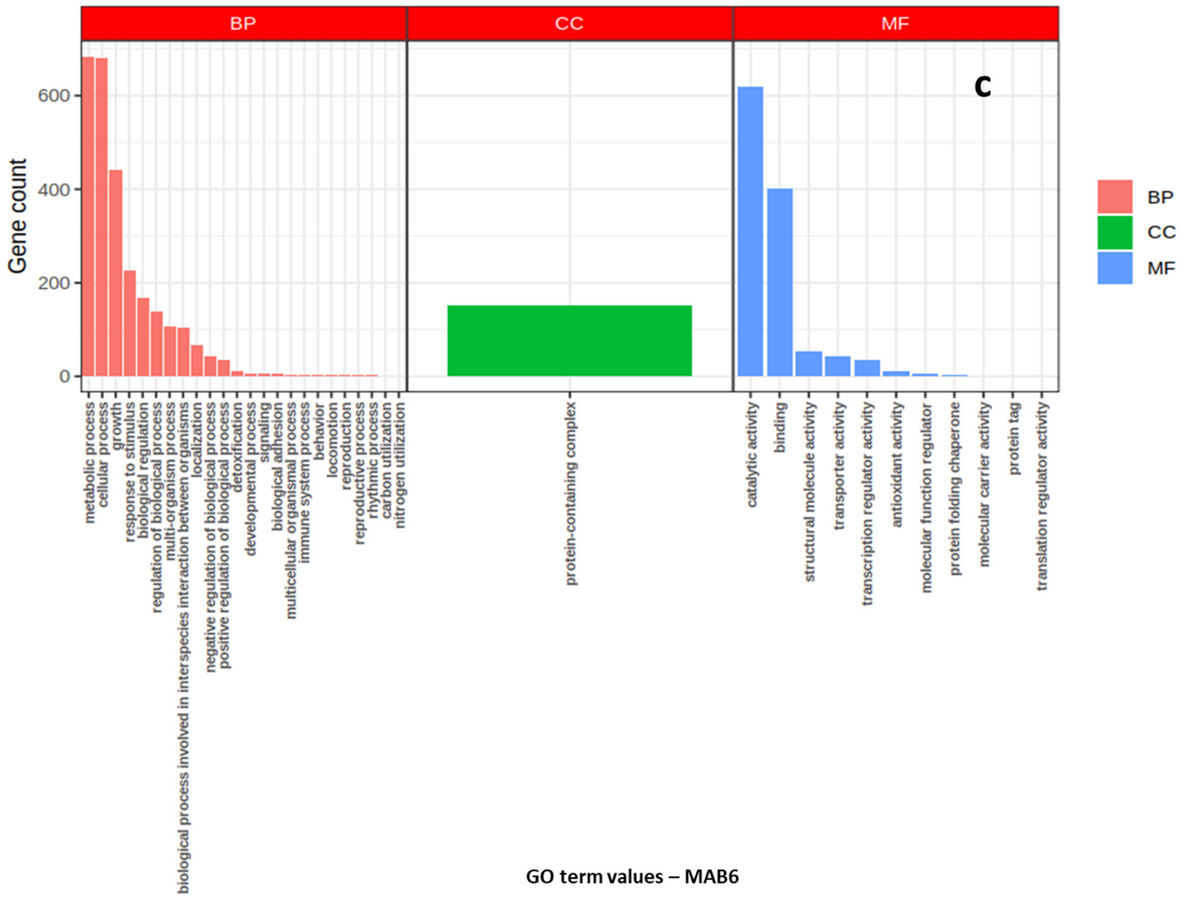

Figure S3 (a), (b) and (c): Plot of the enriched GO terms: Biological process (BP), Cellular components (CC) and Molecular function (MF).
